# LC–MS/MS determination and pharmacokinetic study of columbianadin in rat plasma after intravenous administration of pure columbianadin

**DOI:** 10.1186/s13065-014-0064-1

**Published:** 2014-11-21

**Authors:** Yan-xu Chang, Chun-peng Wang, Jin Li, Yang Bai, Qian Luo, Jun He, Bing Lu, Tao Wang, Bo-li Zhang, Xiu-mei Gao

**Affiliations:** Tianjin State Key Laboratory of Modern Chinese Medicine, Tianjin University of Traditional Chinese Medicine, Tianjin, 300193 China; Tianjin Key Laboratory of Phytochemistry and Pharmaceutical Analysis, Tianjin University of Traditional Chinese Medicine, Tianjin, 300193 China

**Keywords:** Radix angelicae pubescentis, Columbianadin, LC-MS/MS, Intravenous administration

## Abstract

**Background:**

Columbianadin, one of the active coumarins, is isolated from *Radix Angelicae pubescentis* which has been used as a traditional Chinese medicine for the treatment of rheumatic diseases for thousands of years. A fast and sensitive method is required for the determination of columbianadin for pharmacokinetic studies. Liquid chromatography–tandem mass spectrometry (LC-MS/MS) method is a preeminent analytical tool for rapid biomedical analysis.

**Results:**

A sensitive LC-MS/MS method has been validated to determine the concentration of columbianadin in rat plasma after intravenous administration of columbianadin (1, 2.5 and 5 mg kg^−1^). Liquid-liquid extraction was used to extract columbianadin from the rat plasma. Bergapten was selected as an internal standard (IS). The separations were performed on an Eclipse plus C18 column (4.6 × 100 mm, 1.8 μm) with ammonium acetate aqueous solution (1 mmol L^−1^) and acetonitrile as the mobile phase. The flow rate was set at 0.300 mL min^−1^. Quantification was performed using multiple reaction monitoring (MRM) mode to monitor transitions of m/z 329.3 → 229.3 for columbianadin and m/z 217.2 → 202.2 for IS at positive ionization mode. The calibration curve was linear over the concentration range of 4–20000 ng mL^−1^ with a correlation coefficient (r) of 0.996 or better. The precision of intra- and inter-batch assays ranged from 4.02 to 7.33% and accuracies determined at three concentrations ranged between 91.9% and 106%. The lower limit of quantification was about 4 ng mL^−1^.

**Conclusion:**

The proposed LC-MS/MS method is simple, rapid and highly sensitive so that it could be used to evaluate pharmacokinetic properties of columbianadin in rat plasma after intravenous administration.

## Background

*Radix Angelicae pubescentis,* a famous traditional Chinese medicine, is used to treat musculoskeletal, waist and knee pains, cold, dampness and headache [1]. Columbianadin, which is an active coumarin, was selected as one of the markers to evaluate the quality control of *Radix Angelicae pubescentis* [2]. Recently, columbianadin was reported to exhibit anti-inflammatory as well as analgesic activities in mice *in vivo* [3]. It could reduce iNOS in activated cells stimulated with bacterial lipopolysaccharide [4-6]. It has also been reported that columbianadin has calcium channel blocking activity [7,8] and an automated Ca^2+^ uptake using GH4C1 cells *in vitro* [9,10]. Columbianadin has significant bioactivity to inhibit rat platelet aggregation induced by ADP *in vitro.* In addition, columbianadin showed obvious antitumor activity that inhibited human nasopharyngeal carcinoma cell line KB, human leukemia cell line HL-60 [11] and human bladder carcinoma cell line E-J cells *in vitro* [12]. The biological mechanism of columbianadin has received extensive interest and investigation. It was demonstrated that columbianadin could be absorbed into bloodstream by the intestinal absorption and transportation of columbianadin after the pass intestine perfusion [13].

Currently, only a few pharmacokinetic studies have been conducted for the determination of columbianadin in rat plasma. A high-performance liquid chromatography (HPLC) with UV detection has been developed for determination of columbianadin in rat plasma with a lower limit of quantification of 0.10 μg mL^−1^ [14]. However, this method needs large volumes of organic solvent, while having low sensitivity. Compared with traditional HPLC analysis, liquid chromatography- tandem mass spectrometry (LC-MS/MS) methods might provide significant improvements in terms of method sensitivity.

The purpose of the present study was to develop and validate a highly sensitive, rapid and specific method based on LC-MS/MS for the quantification of columbianadin in rat plasma. The method was successfully used for the pharmacokinetic study of columbianadin in rats after intravenous administration.

## Results and discussion

### Optimization of the chromatographic conditions

In terms of resolution and sensitive signal of analyte, several factors including the mobile phase, type of column, gradient elution type and flow rate were optimized. Finally, ammonium acetate aqueous solution (1 mmol L^−1^) and acetonitrile were optimized as the mobile phase when the separation was performed on an Eclipse plus C18 column (4.6 × 100 mm, 1.8 μm). Basing on this condition, the shorter chromatographic time, peak sharpness and sensitive signal were obtained for columbianadin and IS. It was found that ammonium acetate, which was added to mobile phase, could significantly improve the ESI efficiency and enhance response of columbianadin. This result was consistent with previous studies [15].

### Method validation

#### Specificity and sensitivity

**Figure 1:**
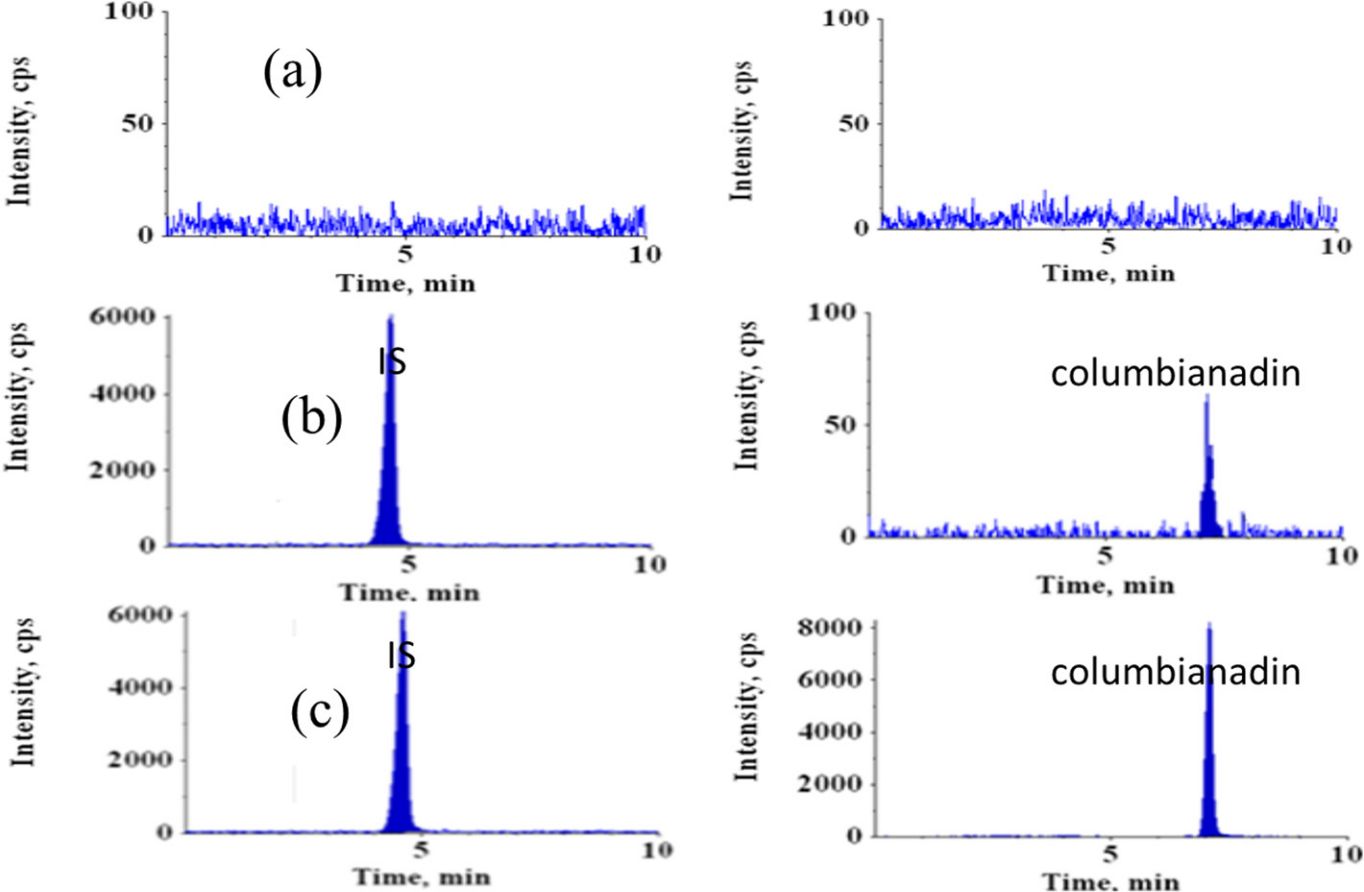
**Representative MRM chromatograms of the columbianadin and IS: (a) blank plasma sample, (b) blank plasma sample spiked the columbianadin at LLOQ, and (c) plasma sample at 30 min after intravenous administrated of 5 mg kg** ^**−1**^ **columbianadin.**

Representative chromatograms of blank plasma, plasma spiked with columbianadin and internal standard (IS) and real plasma sample after intravenous administration of columbianadin, respectively, are given in Figure 1. It was observed that the analyte was well separated and no interferences were detected from endogenous substances or metabolites at the retention time of columbianadin or IS. The lower limit of quantification (LLOQ) of columbianadin in rat plasma was 4 ng mL^−1^ and the RSD was 2.3%. The limit of detection of columbianadin in rat plasma was 1.6 ng mL^−1^. Compared with a previous HPLC method [14], the new LC-MS/MS method might significantly improve sensitivity of determinations.

#### Linearity

In order to obtain a good linear regression analysis of columbianadin, the method was operated by plotting the peak area ratio of columbianadin to IS (Y) versus analyte concentration (X) in spiked plasma samples. The calibration curves showed good linearity over the concentration range of 4-20000 ng mL^−1^. The regression equation was as follows: Y = 0.0243X + 0.0898 (r^2^ = 0.9962). It exhibited a good linearity of columbianadin.

#### Accuracy and precision

The accuracy and precision of inter- and intra-batches were assessed by analyzing six replicate QC samples for each concentration of 20, 1000 and 10000 ng mL^−1^ respectively. The data is presented in Table 1. It can be seen that the RSDs of inter- and intra-batches were below 7.73%, and accuracies of the intra- and inter- batch assays ranged from 91.9% to 106%. The results of intra- and inter-batch analysis indicated that the established method was accurate and reliable.

**Table 1:** **Recoveries, matrix effects, stability and intra-batch, inter-batch accuracy and precision of the columbianadin (n =6)**

**Compound**	**Concentration(ng mL** ^**−1**^ **)**	**Recovery**		**Matrix effect**		**Freeze-thaw cycles**		**At −20°C for 2 weeks**		**Autosampler for 24 h**		**Intra-batch**		**Inter-batch**	
		**Mean**	**RSD**	**Mean**	**RSD**	**Remains**	**RSD**	**Remains**	**RSD**	**Remains**	**RSD**	**Accuracy**	**RSD**	**Accuracy**	**RSD**
		**(%)**	**(%)**	**(%)**	**(%)**	**(%)**	**(%)**	**(%)**	**(%)**	**(%)**	**(%)**	**(%)**	**(%)**	**(%)**	**(%)**
	20	102	9.86	99.7	11.9	97.3	8.85	110	5.81	97.3	12.3	91.9	4.02	92.9	4.29
	1000	85.5	8.71	85.6	5.66	102	11.1	103	5.37	90.0	6.24	104	4.73	106	6.09
	10000	81.4	6.19	90.9	9.20	101	7.04	92.6	6.46	99.2	5.62	106	7.2	98.6	7.73

#### Recovery, matrix effect and stability

Liquid–liquid extraction with ethyl acetate was employed to extract eight coumarins from rat plasma in our previous research [16]. Because the chemical structure of columbianadin is similar with those of the eight coumarins, the liquid–liquid extraction with ethyl acetate was also selected to extract columbianadin from rat plasma. Bergapten was selected as the IS to determine columbianadin in rat plasma after intravenous administration. The extraction recoveries of columbianadin were assessed by analyzing six replicate QC samples at three different levels. The results are shown in Table 1. It can be seen that the mean extraction recoveries of columbianadin at three different levels were within the range of 81.4-102% and RSDs were within the range of 6.19-9.86% for extraction recoveries. The recovery and RSD for extraction recoveries of IS were 94.9% and 3.07%, respectively. It was concluded that liquid-liquid extraction method with ethyl acetate could be used to extract columbianadin from the rat plasma.

The matrix often processed significant interference between the analyte and the endogenous co-eluents and affected the accuracy of analysis results. In this study, the matrix effect of columbianadin in rat plasma (Table 1) ranged from 85.6% to 99.7% at the three level QC samples. The RSDs of the matrix effects were all less than 12%. The above results indicated that there was no ion suppression or enhancement for columbianadin interfered from the rat plasma matrix.

QC samples of columbianadin at three concentrations (20, 1000 and 10000 ng/mL) were used for stability experiments. Results of freeze/thaw stability, autosampler stability and long-term stability are shown in Table 1. It was found that the remains of columbianadin were within the range of 97.3-102%, 92.6-110% and 90.0-99.2% at three QC levels following freeze-thaw cycles, at −20°C for 2 weeks and at autosampler for 24 h, respectively. These results indicated that the stability of QC samples at −20°C for 2 weeks, freeze/thaw stability and post-preparative stability at room temperature for 24 h were acceptable.

#### Pharmacokinetic study

**Figure 2:**
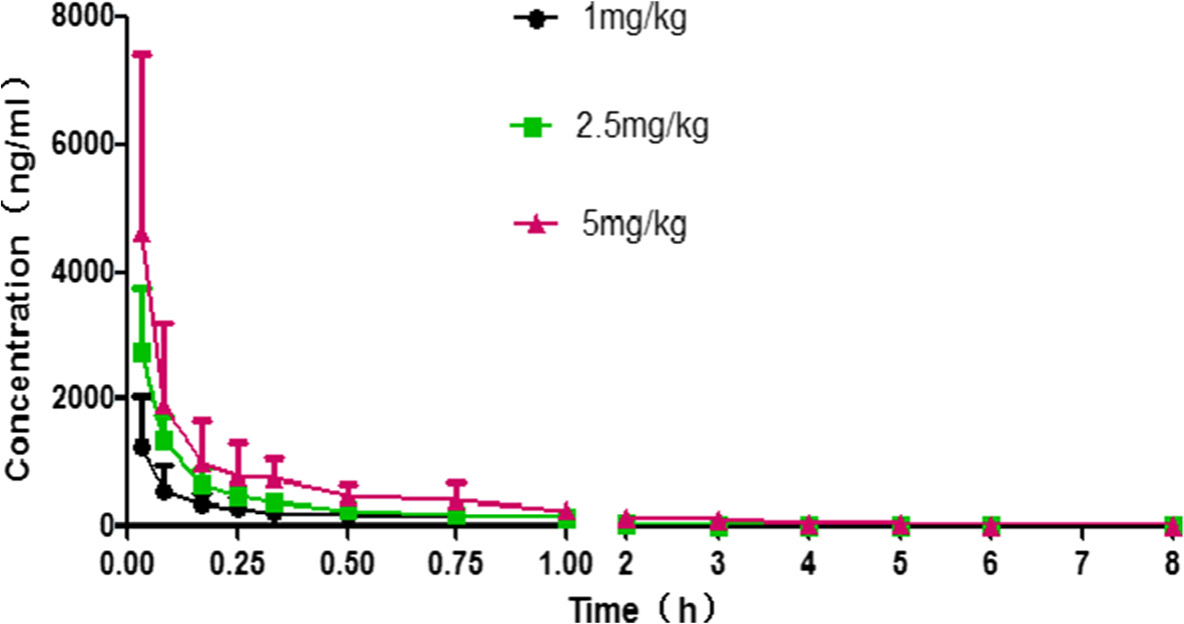
**Plasma concentration-time profiles of the columbianadin in rats after three intravenous dose of 1, 2.5 and 5 mg kg** ^**−1**^ **.**

The established LC-MS/MS method was applied to pharmacokinetic studies following the intravenous administration of columbianadin at the dose of 1, 2.5 and 5 mg kg^−1^, respectively. The plasma concentration–time profile of columbianadin in rats is shown in Figure 2. It was found that the two-compartment model was the best fit pharmacokinetic model to estimate the pharmacokinetic parameters. The pharmacokinetic parameters of columbianadin are listed in Table 2. The distribution half-life(T_1/2α_) were 0.027 ± 0.016, 0.060 ± 0.065 and 0.028 ± 0.023 h after intravenous dose of 1, 2.5 and 5 mg kg^−1^ columbianadin, respectively. These results showed that columbianadin was quickly distributed in the body tissues after intravenous administration of columbianadin. The elimination half-lives (T_1/2β_) of columbianadin were 0.58 ± 0.20, 0.52 ± 0.25 and 0.52 ± 0.22 h after the dose of 1, 2.5 and 5 mg kg^−1^, which demonstrated that the elimination of columbianadin was relatively quick in rats. These results were higher than the elimination half-life time (20 min and 29 min) after the dose of 10 and 20 mg kg^−1^. After intravenous administration of columbianadin at three levels of doses, C_max_ and AUC of columbianadin were mainly dependent on the dose with the coefficient of correlation of 0.986 and 0.920 in the three groups after intravenous administration, respectively, indicating a dose proportionality of columbianadin after intravenous administration. These results were consistent with previous results that there exists a good dose–effect relationship of columbianadin in rat body [14]. It was demonstrated that the present method based on LC-MS /MS with a LLOQ at 4 ng mL^−1^ was sensitive enough for the pharmacokinetics research and could satisfy the requirements of the pharmacokinetics study on columbianadin after intravenous administration.

**Table 2:** **Main pharmacokinetic parameters of columbianadin in rats after intravenous three doses of 1, 2.5 and 5 mg kg** ^**−1**^ **, respectively**

**Parameters**	**Low**	**Media**	**High**
T_1/2α_(h)	0.027 ± 0.016	0.060 ± 0.065	0.028 ± 0.023
T_1/2β_(h)	0.58 ± 0.20	0.52 ± 0.25	0.52 ± 0.22
C_max_(μg/L)	1253 ± 809	2741 ± 1005	4653 ± 2628
AUC_(0-tn)_(μg/Lh)	459 ± 346	563 ± 124	1294 ± 513
AUC_(0-∞)(_μg/Lh)	478 ± 365	593 ± 113	1344 ± 529
MRT_(0-tn)(_h)	1.03 ± 0.19	0.65 ± 0.10	1.22 ± 0.61
MRT_(0-∞)_(h)	1.28 ± 0.23	0.85 ± 0.25	1.62 ± 0.89

### Experimental

#### Chemicals and reagents

Acetonitrile (Dikma technologies Inc, USA) and methanol (Tianjin concord Science Co. Ltd., Tianjin, China) were of chromatographic grade. Columbianadin and bergapten (IS) were purchased from National Institute of the Control of Pharmaceutical and Biological Products (Beijing, China). Ethyl acetate and ammonium acetate were of analytical grade. Deionized water was purified with a Milli-Q Academic ultra-pure water system (Millipore, Milford, MA, USA), and all other reagents were of analytical grade (Tianjin Concord Science Co. Ltd., Tianjin, China).

#### Preparation of stock solution, calibration samples and quality control (QC) samples

The standard stock solutions were prepared by dissolving appropriate quantities of columbianadin and IS in amber volumetric flasks to achieve a concentration of 2.0 mg mL^−1^ and 100 ng mL^−1^, respectively. The calibration curves of columbianadin were prepared at the concentration levels of 4, 8, 20, 40, 200, 500, 2500, 10000 and 20000 ng mL^−1^spiking 100 μL blank rat plasma with 10 μL appropriate standard solution of columbianadin and 10 μL IS. Quality control (QC) samples were prepared following the same sample preparation method described above at the concentrations of 20, 1000 and 10000 ng mL^−1^, respectively. The solutions were stored at 4°C until analysis.

#### Preparation of sample

The plasma samples (100 μL) were thawed at room temperature and mixed with 10 μL IS solution (100 ng mL^−1^), then extracted with ethyl acetate (1000 μL) and swirled for 1 min. The supernatant was collected to dryness in a water bath by using nitrogen at 40°C. The residue was dissolved with 100 μL methanol, swirled for 1 min and centrifuged at 14000 rpm for 10 min. 15 μL of the supernatant was injected into the LC–MS/MS system for analysis.

#### Method validation

The newly established LC-MS/MS method was validated in terms of specificity, sensitivity, linearity, accuracy, precision, recovery, matrix effects and stability.

#### Specificity and sensitivity

The specificity of the method was tested in six different sources of blank rat plasma and the corresponding spiked plasma with columbianadin. Each sample was performed by the same preparation procedure and analytical conditions mentioned above to ensure no interference at the retention time of columbianadin and IS.

#### Linearity and LLOQ

The linearity of the newly established method was evaluated by assaying three calibration curves in rat plasma in the range of 4–20000 ng mL^−1^. The calibration curves were achieved by a weighted (1/*x*^2^) least squares linear fitting through the measurement of the peak-area ratios of columbianadin to IS versus columbianadin levels in rat plasma. The acceptance criterion for each back-calculated standard concentration must be within 15% deviation of the nominal value with the exception of LLOQ, for which the maximum acceptable deviation was set at 20%. The correlation coefficient (r) should also be greater than 0.99. The lower limit of quantification (LLOQ) was defined as the lowest concentration in the standard curve at which the signal-to-noise ratio (S/N) was preliminarily larger than five, the relative standard deviation (RSD) (n =6) was within 20%, and the relative error (RE) was within ±20% [17]. The limit of detection (LOD) was defined as the concentration with the signal-to-noise ratio (S/N) was 3:1.

#### Accuracy and precision

The analytical accuracy and precision were evaluated by assaying at three QC levels. Six samples of each concentration were then carried through the sample preparation as described above to analyze intra- and inter- batch (on three consecutive batches) accuracy and precision. The peak-area ratios of analyte to IS were measured. The accuracy and precision were calculated by the percentage value of calculated concentration to known concentration and the relative standard deviation (RSD), respectively. For acceptable values, the intra- and inter-batch accuracy should range from 85% to 115%. The precision at each concentration was expected to be lower than 15%.

#### Recovery and matrix effect

The recovery and matrix effects of this method were evaluated at three levels of QC samples (n =6). The recovery of columbianadin was calculated by comparing the peak areas obtained from spiked plasma samples with those of peak areas of the analyte spiked post-treatment at corresponding concentrations. Matrix effects were performed by comparing the mean peak areas of the analyte spiked post-treatment to those of the analyte solutions at corresponding concentrations.

#### Stability

The stability of columbianadin was examined by analyzing replicates (n = 6) of the three levels of QC samples under different conditions. The processed stability was assessed by analyzing samples left in autosampler vials at ambient temperature for 24 h. Freeze–thaw stability was evaluated after three cycles of −20°C to 25°C. Long term stability was evaluated by placing plasma at −20°C for two weeks. Columbianadin was considered to be stable in various conditions with a deviation of less than 15%.

#### Application to a pharmacokinetic study in rats

Male Sprague–Dawley rats (250–280 g) were kept under controlled environmental conditions with access to standard laboratory food and water. The rats were randomly divided into three groups with ten rats in each group to diminish the individual variation. The three groups were given columbianadin intravenously for a dose of 1, 2.5, 5 mg kg^−1^, respectively. The rat blood samples (approximately 0.2 mL) were collected in heparinized 1.5 mL polythene tubes at 0.03, 0.08, 0.17, 0.25, 0.33, 0.5, 0.75, 1, 2, 3, 4, 5, 6 and 8 h from their fossa orbitalis after intravenous administration. Then the samples were centrifuged at 4000 rpm for 10 min at 4°C immediately and the supernatant collected. All the supernatant samples were stored at −20°C and were brought to room temperature before use.

#### Data analysis

The pharmacokinetic parameters were carried out by using two-compartmental analysis for intravenous administration groups of rats following DAS pharmacokinetic software package (version 1.0, Chinese Pharmacological Association, China, Anhui).

## Conclusion

In the study, a rapid, sensitive and accurate LC–MS/MS method was established and validated for the analysis of columbianadin in rat plasma after intravenous administration of columbianadin with a shorter time of 10 min. Compared with a previous HPLC method, one advantage of the established LC–MS/MS method is a green and eco-friendly technique with lower consumption of harmful organic solvents when the large numbers of plasma samples should be analyzed. Another advantage of this method is its high sensitivity with lower limit of quantification of 4 ng/mL as well as wide linearity and high specificity. The validated method was successfully applied to a pharmacokinetic study following intravenous administration of pure columbianadin. The intravenous administration of columbianadin experiment showed that the elimination of columbianadin was relatively quick and fitted with linear pharmacokinetics. It is the first report to evaluate the pharmacokinetics of columbianadin by LC-MS/MS.

## Methods

### LC/MS/MS instrument and analytical conditions

The 1200 Agilent series HPLC and an API 3200 triple quadrupole mass spectrometer equipped with an electrospray ionization source were used (Concord, Ontario, Canada). Data acquisition was performed with Analyst 1.4.2 software (AB MDS Sciex). Separations were achieved using an Eclipse plus C18 (4.6 × 100 mm, 1.8 μm) column coupled with a security guard C18 (2.1 × 12.5 mm, 5 μm) (Agilent Technologies, USA). The mobile phase was composed of acetonitrile - ammonium acetate aqueous solution (1 mM, 80:20, v/v). The flow rate was 0.3 mL min^−1^ in isocratic elution mode for 10 minutes. The injection volume was 15 μL. The temperature of the column was set at 25°C.

Bergapten was selected as an internal standard (IS). The multiple reaction monitoring (MRM) mode was selected to monitor the precursor-to-product ion transitions of m/z 329.3 → m/z 229.3 for columbianadin and m/z 217.2 → m/z 202.2 for bergapten (IS) at positive ionization mode. The optimal instrument parameters of the mass spectrometer were as follows: Curtain gas, 15 psi; collision gas, 5 psi; ionSpray voltage, 5500 V; temperature, 350°C; ion source gas1, 40 psi; ion source gas2, 60 psi. Declustering potential (DP) were set at 81 and 55 V, collision energy (CE) were at 13 and 25 V, entrance potential (EP) at 3 and 7 V, the collision cell exit potential (CXP) both at 20 V for columbianadin and IS, respectively.

## Abbreviations

LC-MS/MS: Liquid chromatography coupled with tandem mass spectrometry
RSD: Relative standard deviations
TCMs: Traditional Chinese medicines
MRM: Multiple reaction monitor
ESI: Electrospray ionization
LOD: Limit of detection
LLOQ: Lower limit of quantification
